# The effect of immunoregulation of *Streptococcus lactis* L16 strain upon *Staphylococcus aureus* infection

**DOI:** 10.1186/s12866-017-1038-y

**Published:** 2017-06-02

**Authors:** Maopeng Wang, Shengjie Gong, Shouwen Du, Yilong Zhu, Fengjun Rong, Rongrong Pan, Yang Di, Chang Li, Dayong Ren, Ningyi Jin

**Affiliations:** 1 Key Laboratory of Jilin Province for Zoonosis Prevention and Control, Institute of Military Veterinary, Academy of Military Medical Sciences, Changchun, 130122 People’s Republic of China; 2 College of Food Science and Engineering, Jilin Agricultural University, Changchun, 130118 People’s Republic of China

**Keywords:** *Streptococcus lactis*, *Staphylococcus aureus*, Prophylactic effect, Treatment effect, Th1/Th2 response

## Abstract

**Background:**

*Staphylococcus aureus* is an important pathogen that causes various infections in medical facilities. However, resistance to multiple drugs has made this infection difficult to manage. Thus, new therapeutic strategies are urgently needed to solve this worldwide public health problem. The *Streptococcus lactis* L16 strain was isolated from the fermented hot chili sauce. To explore whether it can be used as a protective agent against *S. aureus* infection, we designed a mouse model of *S. aureus* infection to evaluate the therapeutic potency of *S. lactis*. Mice were grouped into pre-(P) and post-(T) *S. aureus* infection groups following oral administration of *S. lactis* L16. The protection and treatment effects were assessed by examining body weight, internal organ weight, serum cytokines and intestinal secretory IgA alternations.

**Result:**

Oral administration of the *S. lactis* L16 strain reduced the loss of body weight in mice post-infection and alleviated infection-induced hepatomegaly. In particular, the PL16 group (protection with L16) showed more effective resistance to *S. aureus* than the TL16 group (treatment with L16). The level of serum cytokine interferon gamma following oral administration of the L16 strain was remarkably increased during infection, as were interleukin-4 levels during convalescence. The probiotic L16 strain induced more sIgA production than *S. aureus.*

**Conclusion:**

Our data suggest that *S. lactis* L16 is an effective strain with anti-Staphylococcus activity. By regulating the Th1/Th2 response, *S. lactis* can effectively reduce lesions from infection, indicating its therapeutic potential in overcoming antibiotic resistance in this mouse infection model that mimics infections observed in humans.

## Background

*Staphylococcus aureus* is a gram-positive bacterium and a major cause of community- and hospital-acquired bacterial infections [1, 2]. Infection with this bacterium leads to a variety of disease symptoms, such as boils, skin abscesses, endocarditis, and even sepsis*.* Although *S. aureus* is initially sensitive to multiple antibiotics, antibiotics abuse can result in the development of multi-drug-resistant strains, such as methicillin-resistant *S. aureus* (MRSA), vancomycin-intermediate *S. aureus*, and vancomycin-resistant *S. aureus* [3, 4], making *S. aureus* infection difficult to manage.

Additionally, intestinal carriage of MRSA may increase the risk of MRSA infection in children hospitalized in the neonatal intensive care unit [5]. Intestinal colonization of *S. aureus* facilitates nosocomial skin or nasal infection [6, 7]. Preventing intestinal colonization may become a therapeutic method to protect against *S. aureus* transmission. Vaccination is an efficient routine used to inhibit intestinal colonization of pathogens. Although promising, *S. aureus* vaccine development has been impeded due to a lack of sufficiently recapitulated mouse models and a mechanistic understanding of the interaction between *S. aureus* and the human immune system [8, 9].

Microbiota or bacterial vector based therapies are emerging as potential therapeutic strategies [10–12]. The *Streptococcus* genus includes human commensal organisms, and specific species have probiotic characteristics [13, 14]. Immunoregulation is one of the important roles of probiotics. Although the precise mechanism of *S. aureus* immune evasion remains unclear, deregulated T helper cell functions and phagocytic activity are believed to be leading causes of *S. aureus* infection [9, 15]. Therefore, we selected this strain for an in vivo study. Whether *Streptococcus lactis* protects against *S. aureus* in vivo by enhancing the immune response and the immune signalling pathway that can be used to inhibit infection remains unknown. Our previous work isolated the *Streptococcus lactis* strain L16 from the fermented hot chili sauce, and in vitro experiments indicated that L16 retains antibacterial activity and probiotic characteristics. In this regard, we aimed to characterize the inhibitory efficacy of *S. lactis* L16 in vivo in a mouse model. Oral administration of *S. lactis* L16 was used to explore its effectiveness in preventing and treating *S. aureus* infection. Mouse body weight, internal organ weight, level of sIgA and cytokine changes were assessed to evaluate its prophylactic effect.

## Methods

### Experimental design

**Fig. 1:**
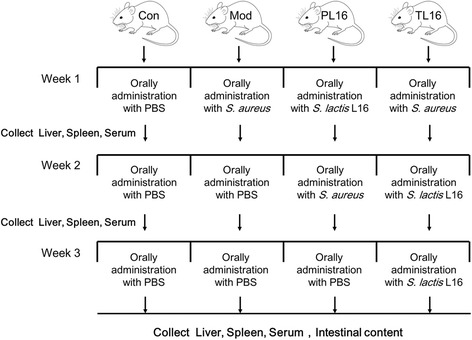
Study design scheme. All of female Kunming mice were fed a normal diet throughout the experimental period. The strains (*S. aureus* CICC 21600 or *Streptococcus lactis* L16) were suspended in PBS and administrated into experimental mice. After pre-exposure L16 for 1 week, mice in the PL16 group were then orally administrated with *S. aureus* for 1 week and with PBS the following week. Mice in the TL16 group were subjected to *S. aureus* infection for 1 week and treated with L16 the following 2 weeks. In the Mod group, mice were orally infected with *S. aureus* for 1 week. The Con group remained oral administration of PBS instead of *S. aureus* or *S. lactis* L16 for 3 weeks

This experiment involved four groups: control (Con), mouse model (Mod), protection with L16 (PL16) and treatment with L16 (TL16) groups. All mice were provided abundant food and water. Mice in the Con group were orally administered phosphate buffer every day, and those in the Mod group were subjected to oral administration of *S. aureus* daily for 1 week. Mice in the PL16 group were orally administered *S. lactis* L16 and orally infected with *S. aureus*. *Streptococcus lactis* L16 was administered for intervention with post-infection of *S. aureus* for 1 week in the TL16 group. Schematics of the experimental design are shown in Fig. 1.

### Mice

Female Kunming mice (age 5 weeks, weight 18–20 g) were obtained from the Changchun Institute of Biological Products Co. Ltd., Changchun, China. All mice were provided a diet and water ad libitum. They were housed in a quiet, ventilated cage under natural light at a temperature of 20 ± 1 °C and a humidity of 50% ± 10%. After 1 week of adaptation to the feeding protocol, mice were randomly divided into four groups (*n* = 6). All animal handling and experiments were approved by the Academy of Military Medical Sciences Ethics Committee.

### Bacterial strains

The strain *S. lactis* L16 was previously isolated from the fermented hot chili sauce and cultured in de Man, Rogosa, and Sharpe broth (MRS; Qingdao Hope Bio-Technology Co. Ltd., Qingdao, China) at 37 °C for 24 h. Cells were harvested by centrifugation at 7500 rpm for 10 min and then diluted into 10^9^ colony forming units (CFU)/mL with 0.9% sterile saline solution. *Staphylococcus aureus* (CICC 21600) was obtained from the Laboratory of Food Safety of Jilin Agricultural University, Changchun, China. Cells were cultured with a dilution of 10^6^ CFU/mL in Luria-Bertani (LB) broth at 37 °C for 24 h and stored at 4 °C.

### Infection with *S. aureus* in mice

Based on body weight, pathogenic *S. aureus* was administered at 10^7^ CFU/kg according to the method described by Kim et al. [16]. Mice were monitored daily. Initial clinical symptoms were sluggishness, loss of appetite, coarse hair, diarrhoea and tremors. In the Mod group, severe congestion and stench of multiple organs were observed after dissection, while mice in the Con group were asymptomatic.

### Internal organ weight

Mice in each group were weighed and sacrificed by cervical dislocation. Livers and spleens were collected, and the index was calculated as the percentage of fresh liver or spleen weight to fresh body weight [17] as follows:

$$ \mathrm{Liver}\ \mathrm{and}\ \mathrm{spleen}\ \mathrm{index}=\left[\mathrm{weight}\ \mathrm{of}\ \mathrm{liver}\ \mathrm{or}\ \mathrm{spleen}\ \left(\mathrm{g}\right)/\mathrm{body}\ \mathrm{weight}\ \left(\mathrm{g}\right)\right]\times 1000. $$

### Interferon gamma (IFN-γ), interleukin-4 (IL-4) and intestinal sIgA

Blood was collected from the orbital cavity. Mice were anaesthetized with 200 mg/kg amylobarbitone by abdominal injection. Serum was obtained by centrifugation at 3500 rpm for 10 min as described previously [18]. Intestinal lavage from a 1-cm colon sample using PBS was collected and stored at −80 °C [19]. The levels of cytokines (IFN-γ and IL-4) in the serum and sIgA in intestinal fluid (in week 3) were quantified by ELISA [20–24].

### Statistical analysis

All data are presented as the mean ± standard deviation (SD). Statistical analyses were performed using GraphPad Prism 6.0 software (GraphPad Software, Inc., La Jolla, CA, USA). Cytokine data were analysed and compared between groups by one-way analysis variance followed by Dunnett’s test [25]. A *p*-value <0.05 was considered to indicate statistical significance.

## Results

### Detection of weight change associated with *S. aureus* infection

**Fig. 2:**
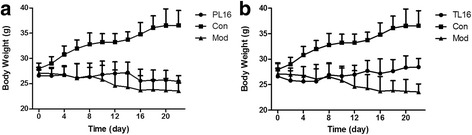
Changes in body weight. **a** Protection group (PL16): Oral administration of L16 for 1 week (week 1) and continued feeding with *S. aureus* the following week (week 2). Normal feeding during the last week (week 3). **b** Treatment group (TL16): Oral administration of *S. aureus* for 1 week and continued feeding with L16 the following 2 weeks. The control group (Con): Oral administration of PBS instead of *S. aureus* or *S. lactis* L16 for 3 weeks. All groups were fed a normal diet throughout the experimental period. Data are presented as the mean ± SD (*n* = 2)

Body weight was monitored every 2 days. Body weight changes were shown in Fig. 2. Compared with the Con group, infection with *S. aureus* led to a loss of weight. Mice in both the PL16 and TL16 groups maintained a stable weight for at least 3 weeks. Although a slight decrease in mean weight was observed in the PL16 group compared with the TL16 group, the mean weight of both groups was approximately 25 g, with no significant difference.

### Detection of mouse internal organ weight

Mice in the PL16 group were orally administered *S. lactis* L16 for 1 week and fed *S. aureus* the following week. Mice were fed a normal diet the last week. Mice in the TL16 group were fed *S. aureus* for 1 week and fed *S. lactis* L16 the following 2 weeks. The control group (Con) was fed a normal diet throughout the experimental period. Data are presented as the mean ± SD (*n* = 4).

Internal organ weight data are shown in Table 1. Mice who received oral administration of *S. lactis* (PL16 group) and were infected with *S. aureus* exhibited a similar index to the Con group. The liver index of mice in the TL16 group was 18% higher than that of the Con group (*p* < 0.05) post-infection with *S. aureus*. After intervention therapy for 1 week, the index decreased and was 8% higher than that of the PL16 group.

**Table 1:** The ratios of organs to body weight during infection

	Week 1		Week 2		Week 3	
	Liver	Spleen	Liver	Spleen	Liver	Spleen
Con	48.15 ± 0.17	4.61 ± 0.99	48.61 ± 1.99	4.55 ± 1.12	47.87 ± 5.65	4.62 ± 0.41
PL16	48.26 ± 0.18	3.54 ± 1.54	48.49 ± 1.88	3.44 ± 1.02	42.91 ± 5.32	3.61 ± 0.88
TL16	54.35 ± 2.80	4.75 ± 0.86	52.65 ± 1.05	4.98 ± 1.50	46.56 ± 9.01	5.65 ± 0.81
Mod	58.18 ± 1.14**	4.67 ± 1.78	56.18 ± 5.37**	4.76 ± 1.83	51.77 ± 4.70	5.06 ± 0.97

The data is presented by ratio of organ to body weight by mean ± SD. (**, *p* < 0.05).

Groups are matched with experimental design in Method section. Con: the control group with orally administration of PBS. PL16: the protection group with administration of L16 strain before *Staphylococcus aureus* infection. TL16: the treatment group with administration of L16 strain after *Staphylococcus aureus* infection. Mod: murine model group with *Staphylococcus aureus* infection daily for 1 week.

### Detection of serum IFN-γ and IL-4

**Fig. 3:**
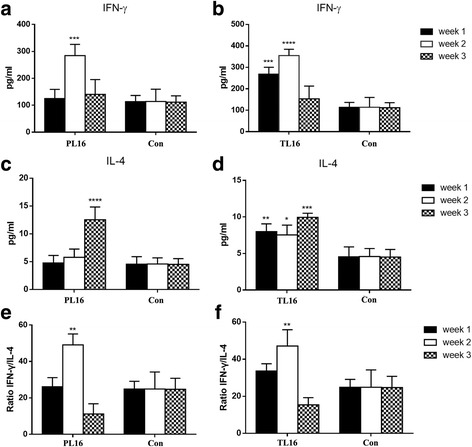
Concentration and ratio of serum cytokines in mice. Concentration of serum IFN-γ (**a**) and IL-4 (**c**) in the PL16 group. Concentration of serum IFN-γ (**b**) and IL-4 (**d**) in the TL16 group. Ratio of IFN-γ to IL-4 in the PL16 (**e**) and TL16 (**f**) groups. *p < 0.05, **p < 0.01, ***p < 0.001, and ****p < 0.0001 compared with Con. L16: *S. lactis*, Con: control group. Data are presented as the mean ± SD

The serum levels of IFN-γ and IL-4 over the 3-week experimental period are shown in Fig. 3. The expression of IFN-γ was significantly increased (*p* < 0.001) during infection in both the PL16 (Fig. 2a) and TL16 (Fig. 2b) groups, while IL-4 remained stable. During convalescence, IFN-γ significantly increased (*p* < 0.001) and IL-4 decreased (*p* < 0.001). Further analysis revealed that the ratio of IFN-γ to IL-4 significantly decreased during infection and during the recovery phase.

### Detection of sIgA in the colon content

**Fig. 4:**
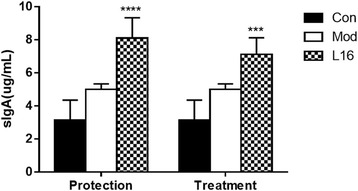
Final concentration of sIgA in intestinal fluid. ELISA was used to detect sIgA in intestinal fluid. Con: control group, Mod: mice infected with *S. aureus* without *S. lactis* administration. L16: *S. lactis*. *p < 0.05 compared with Con

The level of sIgA in the colon content was shown in Fig. 4. The secretion of sIgA following L16 administration was 2-fold higher in the PL16 and TL16 groups than in the Con group (*p* < 0.05). Intestinal sIgA secretion was modestly higher in protection than in treatment, which was consistent with clinical symptoms [26]. However, oral administration of L16 elevated intestinal sIgA in both the PL16 and TL16 groups to a greater extent than in the Con and Mod groups.

## Discussion

*Staphylococcus aureus* is a major source of community- and hospital-acquired bacterial infections [27]. It is also a leading cause of morbidity and mortality in medical facilities. It frequently colonizes human skin and mucosa and conditionally causes a wide range of infections, leading to various clinical symptoms, including boils, impetigo, arthritis and sepsis. *Staphylococcus aureus*, an important opportunistic pathogen, causes a variety of infectious diseases [28, 29]. It has become prevalent because of the use of drugs, such as antibiotics, and the development of MRSA. Several new therapies are under investigation to replace antibiotics therapy [30]. Thus, a novel vaccine or therapy is urgently needed to solve this worldwide public health problem [31]. A microbiome-based therapeutic strategy, which may play a therapeutic role in a variety of infectious diseases, has received growing recognition [32, 33]. Microbiota transplantation has been applied for several years to antagonize infectious diseases, such as recurrent *Clostridium difficile* [34]. However, whether one or several species-specific strains play a vital role against *S. aureus* infection that can be developed into a novel treatment therapy remains unclear.

It is important to note that *S. lactis* inhibits the growth of *S. aureus* in vitro [35]. An independent study also showed that an enhanced Th1 response was beneficial to attenuate infection [36]. To our knowledge, the present study is the first to demonstrate that *S. lactis*, an isolate from the fermented food, exhibits inhibitory activity against *S. aureus* by oral administration in a murine infection model. Our results show that feeding *S. lactis* can reduce weight loss induced by *S. aureus* infection in both the PL16 and TL16 groups. The internal organ index was introduced to present liver and spleen pathological characteristics and severity. Reduced severity of pathology was shown in the PL16 group, which indicates that *S. lactis* L16 may have a prophylactic effect on *S. aureus* infection.

We also found that in contrast to serum IL-4, serum IFN-γ increased in the PL16 and TL16 groups during infection and returned to normal during convalescence, consistent with previous observations [37]. Serum IFN-γ represents a cytokine expressed from Th1, an indicator of cellular immunity [38, 39]. Elevated IFN-γ may promote cytotoxic lymphocyte proliferation to eliminate invasive pathogens. After removal, the Th2 response increases to continue the clearance of the surviving pathogen and establishes a memory immune response [40, 41]. Finally, we also evaluated the concentration of sIgA in intestinal fluid. We conclude that L16 causes higher secretion of sIgA in the intestinal tract. In mice, secretory IgA would initially be beneficial to protect the intestine from *S. aureus* invasion [42, 43]. An enhanced Th1 response and increased secretion of sIgA illustrate the protective roles of L16.

In summary, *S. lactis* L16 is a probiotic with potent anti-Staphylococcus activity in vivo. It promotes IFN-γ and IL-4 secretion and stimulates the Th1 response at the early phase of disease development by enhancing the phagocytosis of pathogens, thus preventing further harm from infection, which is synergistically supported by the upregulation of sIgA. These results imply the therapeutic anti-infection potential of L16 for future human clinical applications.

## Conclusion

This study shows *S. lactis* L16, an isolated from the fermented hot chili sauce, owns a potentially immunoregulatory role on protecting mice against pathogenic *S. aureus*. Besides, the changes of cytokines and sIgA seems to be one of important factors to improve clinical symptons in a murine model. Therefore, this probiotic strain L16 may be used as a potential and promising therapeutic drug to prevent or treat *S. aureus*.infection in the gastro-Intestinal tract.

## Abbreviations

IFN-γ: Interferon-gamma
IL-4: Interleukin-4
MRSA: Methicillin-resistant *S. aureus*
sIgA: Secretory immunoglobulin A
Th1/2: Type 1/2 helper cell
